# EFFECTIVENESS VS. EFFICACY: CREATING EVIDENCE FROM GERIATRIC SURGERY QUALITY PROGRAMS

**DOI:** 10.1093/geroni/igz038.1487

**Published:** 2019-11-08

**Authors:** Meixi Ma

**Affiliations:** American College of Surgeons, Chicago, Illinois, United States

## Abstract

The American College of Surgeons National Quality Improvement Program started a “Geriatric Pilot” in January 2014. This initiative has collecting specialty variable related to older adults in more than 60,000 patients 65 years and older undergoing operations. Multiple research publications have been generated from this pioneering national quality program focused on surgical quality in older adults. The purpose of this presentation will be to review the major findings of this new body of research. Studies have focused on functional trajectory following operations, postoperative delirium, and peri-operative decision-making.

